# Amphiregulin enhances intercellular adhesion molecule-1 expression and promotes tumor metastasis in human osteosarcoma

**DOI:** 10.18632/oncotarget.5679

**Published:** 2015-10-19

**Authors:** Ju-Fang Liu, Ya-Ting Tsao, Chun-Han Hou

**Affiliations:** ^1^ Central Laboratory, Shin-Kong Wu Ho-Su Memorial Hospital, Taipei, Taiwan; ^2^ Department of Orthopedic Surgery, National Taiwan University Hospital, Taipei, Taiwan

**Keywords:** amphiregulin, osteosarcoma metastasis, cell migration, ICAM-1, EGFR

## Abstract

Osteosarcoma is a common, high malignant, and metastatic bone cancer. Amphiregulin (AREG) has been associated with cancer cellular activities. However, the effect of AREG on metastasis activity in human osteosarcoma cells has yet to be determined. We determined that AREG increases the expression of intercellular adhesion molecule-1 (ICAM-1) through PI3K/Akt signaling pathway via its interaction with the epidermal growth factor receptor, thus resulting in the enhanced cell migration of osteosarcoma. Furthermore, AREG stimulation increased the association of NF-κB to ICAM-1 promoter which then up-regulated ICAM-1 expression. Finally, we observed that shRNA silencing of AREG decreased osteosarcoma metastasis *in vivo*. Our findings revealed a relationship between osteosarcoma metastatic potential and AREG expression and the modulating effect of AREG on ICAM-1 expression.

## INTRODUCTION

Osteosarcoma is a malignant neoplasm of the bone, which is prevalent in teenagers and young adults. The cause of osteosarcoma is unclear. However, numerous factors are associated with the increased risk of osteosarcoma including age, genetic inheritance, chronic inflammation, virus infection and radiation exposure [1]. Most patients with osteosarcoma are treated with chemotherapy or radiation therapy. However, some patients remain at high risk of relapse or metastasis, thus necessitating successful treatment [2]. Although surgery remains the vital modality for treating the primary tumor in such patients, adjuvant chemotherapy plays an essential role in controlling sub-clinical metastatic disease [3]. Because osteosarcoma has highly invasive and metastatic potential, determining the factors promoting metastasis of osteosarcoma is imperative [4].

Cancer metastasis is a complicated process involving several attachment and detachment events. Therefore, as expected, numerous studies have revealed a correlation between changes in cell adhesion, tumor invasion and metastasis [5, 6]. For example, loss of adhesion between cells enables malignant tumors cells to detach from the primary tumor and to enter the circulatory system. However, at the later stages of metastasis, expression of adhesion molecules facilitating intercellular contacts is required for efficiently spreading metastatic cells [7–9]. One of these cell adhesion molecules is intracellular adhesion molecule-1 (ICAM-1/CD54), a member of the immunoglobulin supergene family [10–12]. ICAM-1, a surface glycoprotein, is expressed at a low basal level on the surface of leukocytes and endothelial cells. ICAM-1 is also constantly present at low concentrations in fibroblasts, keratinocytes, and epithelial cells, but is up-regulated in response to several inflammatory mediators [13]. Numerous studies have reported that ICAM-1 expression contributes to human tumor progression because elevated expression levels of this protein have been observed in gastric, pancreatic and breast cancer tissues, and the highest expression levels were observed in samples from patients with metastases [14]. Many studies have supported the potential involvement of ICAM-1 in the metastasis of certain tumors. For example, knockdown of ICAM-1 reduced the invasiveness of prostate cancer cells [15] and depletion of ICAM-1 inhibited melanoma lung metastasis [16]. ICAM-1 was also involved in the invasion of breast, lung, gastric, oral, and bone cancer cells [10, 15, 17–19].

In our previous study, we determined that elevated ICAM-1 expression was promoted the metastasis of osteosarcoma: the expression level of ICAM-1 was elevated in two metastatic osteosarcoma cell lines and ICAM-1 siRNA reduced their tumor cell migration (transforming growth factor alpha) [20]. Moreover we suggested that ICAM-1 expression is up-regulated by PI3K/Akt signaling pathway through the epidermal growth factor receptor (EGFR) [20]. The EGFR is a receptor tyrosine kinase of the ErbB family. Upon activation by the binding of specific ligands, the autophosphorylation of the EGFR at several tyrosine residues initiates downstream signal transduction cascades, principally of the mitogen-activated protein kinase, Akt and c-Jun N-terminal kinase pathways. These protein kinases modulate several cellular activities such as cell migration, adhesion, and proliferation. Therefore the aberrant activation of EGFR has been associated with various cancers through several mechanisms including receptor overexpression, mutation, receptor ligand overexpression, and ligand-independent activation [21]. For example, overexpression of Transforming growth factor-alpha (TGF-α), one of the main ligands of the EGFR, has been observed in various of malignant tumors such as kidney cancer, pancreatic cancer, colon cancer and breast cancer [22–25]. Furthermore, we have demonstrated in our previous study that TGF-α mRNA and protein level were higher in osteosarcoma cell lines and that TGF-α promotes osteosarcoma metastasis through its interaction with EGFR [20].

In addition to TGF-α, amphiregulin (AREG) is another EGFR ligand that is known to promote cancer growth and progression. AREG is a member of the epidermal-growth-factor-like family [26, 27]. It can bind to and activate the EGFR through three general mechanisms: autocrine, paracrine, and juxtacrine. Therefore, it can regulate numerous cellular functions such as cell proliferation, survival, migration, differentiation, adhesion and angiogenesis [28–31]. Physiologically, AREG stimulates the proliferation of normal cells such as fibroblasts, keratinocytes, urothelial or epithelial cells [32, 33]. Studies have reported that AREG plays a vital role in the development and maturation of various organs including the mammary glands [34, 35], bones [36], or placenta [37]. Nonetheless, AREG promotes the migration of tumor cells [38, 39] and its overexpression has been observed in several cancer tissues such as colorectal, gastric, pancreatic and breast cancer [40–43]. Moreover a microarray analysis revealed that AREG was up-regulated in metastatic tumors of the liver [44].

To understand the role of AREG in osteosarcoma metastasis, in the current study, we examined its expression level and the effect of exogenous AREG treatment in osteosarcoma cells. We determined that two osteosarcoma cell lines expressed high levels of AREG and that further AREG stimulation enhanced ICAM-1 expression, thus contributing to the increased cell migration of osteosarcoma. Using molecular and pharmacological approaches, we also found that the AREG-induced cell migration of osteosarcoma cells was mediated by EGFR signaling through its downstream PI3K/Akt pathway. Furthermore, increased EGFR signaling enhances the activity of nuclear factor-κB (NF-κB) and the recruitment of NF-κB to the ICAM-1 promoter which subsequently up-regulate ICAM-1 expression. Our findings of the AREG-responsive signaling pathway provides valuable clues for understanding the mechanisms of human osteosarcoma metastasis and present an opportunity to develop highly effective clinical therapies in the future.

## RESULTS

### AREG-induced cell migration in osteosarcoma cell line can be further enhanced by AREG supplementation, but be inhibited by ICAM-1 siRNA

**Figure 1:**
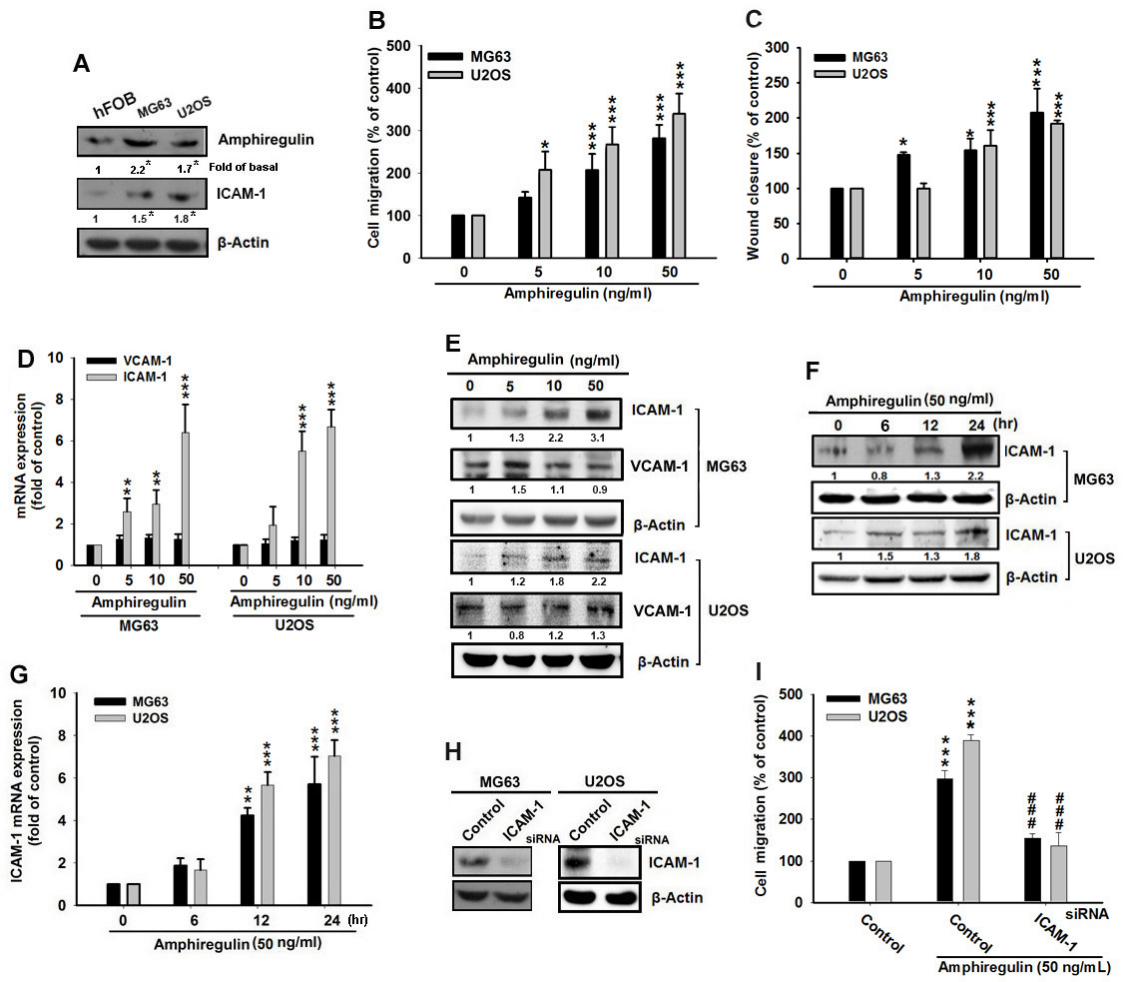
ICAM-1 regulates the AREG-induced cell migration of osteosarcoma **A.** Total protein was extracted from hFOB 1.19, MG63, and U2OS cells and the levels of AREG and ICAM-1 were examined by western blot analysis. **B–C.** Cells were incubated with different concentrations of AREG for 24 hr and *in vitro* migration was measured using the Transwell assay and Wound-healing migration assay. **D–G.** Cells were incubated with various concentrations of AREG for 24 hr or with 50 ng/mL AREG for 6, 12 or 24 hr. The mRNA level or protein expression of ICAM-1 was then measured by qPCR or Western blotting respectively. **H.** Cells were transfected with ICAM-1 or negative control siRNA (Control) for 24 hr, **I.** followed by treatment with AREG for 24 hr. Cell migration was then analyzed using the Transwell assay. All bars represent the mean ± SEM. The asterisks indicate that the data are significantly different from the control without AREG treatment. *represents *P* < 0.05, **represents *P* < 0.01, ***represents *P* < 0.001, as compared to respective control by using one-way ANOVA followed by Bonferroni's post-hoc test. ###represents *P* < 0.01, comparisons to the control treated with AREG by using one-way ANOVA followed by Bonferroni's post-hoc test.

AREG has been shown to increase cell migration and metastasis in various human cancer cells [38, 39, 45]. To determine the clinical significance of AREG in patients with osteosarcoma, we utilized a tissue microarray for evaluation by IHC to compare the expression of AREG in normal bone and different grades of osteosarcoma. Representative examples of IHC staining for AREG in normal bone and osteosarcoma tissues with different grades are shown in Figure S1A–S1B. The expression of AREG had significantly increased with tumor progression (Figure S1A–S1B). Next, to understand the effect of AREG on osteosarcoma cells, we first determined the levels of AREG in two human osteosarcoma cell lines (MG63 and U2OS) and in one human fetal osteoblastic cell line (hFOB 1.19). The levels of AREG were significantly elevated in the MG63 and U2OS cells compared with the low basal level expressed in the hFOB 1.19 cell line (Figure 1A and Figure S1C). This finding is consistent with those of previous studies, suggesting that metastatic osteosarcoma is induced by the elevated AREG expression in the cells. To test this further, we supplied osteosarcoma cells with exogenous human AREG. We applied the Transwell assay to examine the AREG-triggered migration, and determined that AREG treatment further increased the migration of both the MG63 and U2OS cells in a dose-dependent pattern (Figure 1B). Furthermore, AREG treatment increased the wound-healing activities and cell motility of these cell lines [46] (Figure 1C), although it did not affect cellular viability in both human osteosarcoma cell lines (data not shown). To understand the level of AREG between the normal and the malignant cells, we examined the levels of AREG and ICAM-1 between hFOB 1.19 and osteosarcoma (MG63 and U2OS). The level of AREG and ICAM-1 were significantly elevated in MG63 and U2OS cell lines compared with hFOB 1.19 (Figure S1C).

Our study demonstrates that higher expression of AREG promotes the migration of osteosarcoma cells and that AREG supplementation can further enhance migration. Because recent studies have also indicated that ICAM-1 plays a key role in cancer cell migration and invasion [47, 48], ICAM-1 may be involved in the AREG-induced migration. Therefore, we measured the expression levels of ICAM-1 mRNA and protein in AREG treated osteosarcoma cells and determined that these levels were increased by AREG treatment in a dose-dependent and time-dependent pattern (Figure 1D–1G). However, AREG treatment had no effect on the mRNA or protein level of VCAM-1 (vascular cell adhesion molecules) (Figure 1D–1G), though these molecules have also been shown to influence cancer invasion [49]. We also found that the expression of ICAM-1 was elevated in osteosarcoma cells (Figure 1A). To further confirm the role of ICAM-1 in the AREG-induced migration, the MG63 and U2OS cells were transfected with ICAM-1 small interfering RNA (siRNA) for 24 hr. Transfection of ICAM-1 siRNA reduced the protein level of ICAM-1 (Figure 1H), also in addition to fully suppressing the AREG-induced cell migration (Figure 1I). These observations imply that enhanced ICAM-1 expression contributes to the AREG-induced cancer cell migration and ICAM-1 works downstream of AREG to regulate the cell migration of osteosarcoma.

### AREG mediates the cancer cell migration of osteosarcoma through EGFR

**Figure 2:**
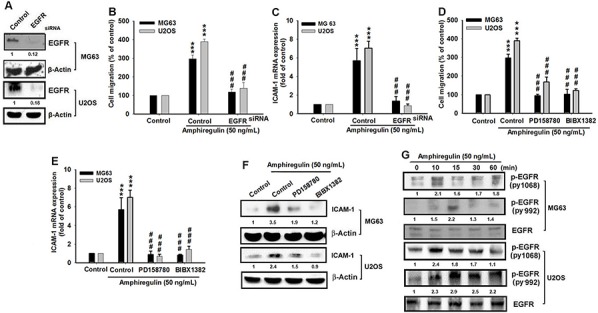
EGFR is involved in AREG-mediated migration of human osteosarcoma cells **A.** Cells were transfected with EGFR siRNA or negative control siRNA (Control) for 24 hr. The EGFR expression was examined by western blotting. **B–C.** After transfection of siRNA, cells were treated with AREG for 24 hr. Cell migration was analyzed using the Transwell assay and the mRNA level of ICAM-1 was measured. **D–F.** Cells were pretreated for 30 min with PD158780 (5 μM) or BIBX1382 (10 μM) followed by the stimulation with AREG for 24 hr. Both EGFR tyrosine kinase inhibitors can suppress the AREG-induced cell migration and the AREG-enhanced expression of ICAM-1 in mRNA or protein level. **G.** Cells were incubated with AREG for indicated time intervals and EGFR phosphorylation at Y992 or Y1068 was detected with specific antibodies. All bars represent the mean ± SEM. The asterisks indicate that the data are significantly different from the control without AREG treatment. ***represents *P* < 0.001, as compared to respective control by using one-way ANOVA followed by Bonferroni's post-hoc test. ###represents *P* < 0.01, comparisons to the control treated with AREG by using one-way ANOVA followed by Bonferroni's post-hoc test.

Several studies have reported that AREG specifically binds to the EGFR, which affects several cellular functions such as cell proliferation, differentiation and migration [41, 50, 51]. In addition, the EGFR plays a critical role in cancer cell migration and invasion [52]. To test whether AREG increased the cell migration of osteosarcoma through EGFR, we reduced the EGFR expression by transfecting EGFR siRNA (Figure 2A) and found that EGFR siRNA inhibited the AREG-induced cancer cell migration and inhibited the AREG-induced ICAM-1 upregulation of the mRNA level (Figure 2B–2C). Furthermore, treatment with PD158780 and BIBX1382, two commonly used EGFR tyrosine kinase inhibitors that can block the autophosphorylation (activation) of EGFR [53, 54], had the same suppressive effects of EGFR siRNA on the AREG-enhanced migration and ICAM-1 upregulation, indicating that EGFR activation is required for AREG-mediated migration (Figure 2D–2F). Because activating the EGFR leads to the autophosphorylation of its tyrosine residues [55–57], we examined the level of the phosphorylated EGFR at tyrosine 1068 and 992 after treatment. We observed that AREG treatment increased the level of phosphorylated EGFR (Figure 2G). These results indicated that AREG and EGFR interacted to regulate the migration of osteosarcoma and the expression level of ICAM-1.

### PI3K/Akt signaling pathway is involved in AREG-mediated ICAM-1 up-regulation and cell migration of osteosarcoma cells

**Figure 3:**
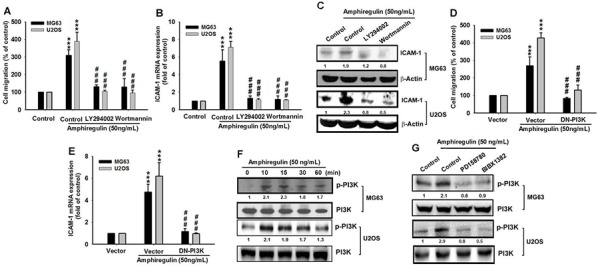
PI3K signaling regulates the AREG response in human osteosarcoma **A–C.** Cells were pretreated with 0.1% DMSO as control, LY294002 (10 μM) or Wortmannin (1 μM) for 30 min followed by the stimulation with AREG for 24 hr. Cell migration and ICAM-1 expression level were examined. **D–E.** As PI3K inhibitors, the dominant-negative mutation of PI3K (DN-PI3K) had the same suppressive effect on both the AREG-induced cell migration and ICAM-1 up-regulation. Results are shown as the mean ± SEM. The asterisks indicate *t*-test comparisons to the control without AREG treatment. ***represents *P* < 0.001. ##represents *P* < 0.01 for one-way ANOVA comparisons as indicated. **F.** Cells were treated with AREG (50 ng/ml) for the indicated times and PI3K p85 phosphorylation (p-PI3K) was detected by specific antibody. **G.** Cells were pretreated with 0.1% DMSO as control, PD158780 (5 μM) or BIBX1382 (10 μM) for 1 hr followed by stimulation with AREG for 10 min and then the level of phosphorylated p85 (p-PI3K) was examined.

Previous studies have demonstrated that EGFR overexpression promotes cancer cell survival, proliferation, migration, and invasion [58]. The promotion mechanism is associated with the PI3K/Akt signaling pathway that stimulates cell survival and suppresses cell apoptosis [59, 60]. Because AREG supplementation can increase EGFR activity, which consequently enhances osteosarcoma cell migration, we speculated whether the PI3K/Akt signaling pathway mediates the AREG-induced cell migration. To test this, we first treated osteosarcoma cells with PI3K inhibitors, Ly294002 and Wortmannin [61]. We found that treatment with PI3K inhibitors suppressed the AREG-induced migration and ICAM-1 expression in mRNA and protein level (Figure 3A–3C). The same suppressive effect was observed in the dominant-negative mutant of PI3K p85-transfected cells (Figure 3D–3E), indicating that PI3K works downstream of AREG to affect the migration of osteosarcoma and to regulate the expression of ICAM-1. To further explore whether AREG actives PI3K, we measured the level of phosphorylated p85, which has been associated with PI3K activity [62]. We observed that AREG treatment led to an increase in the phosphorylation of p85 (Figure 3D). These reading suggest that AREG treatment increases the activation of the PI3K pathway through EGFR signaling. Therefore, we decreased EGFR signaling by treatment with EGFR inhibitors, PD158780 or BIBX1382, and measured the level of phosphorylated p85. Both inhibitors were found to suppress the AREG-induced PI3K activation (Figure 3E).

**Figure 4:**
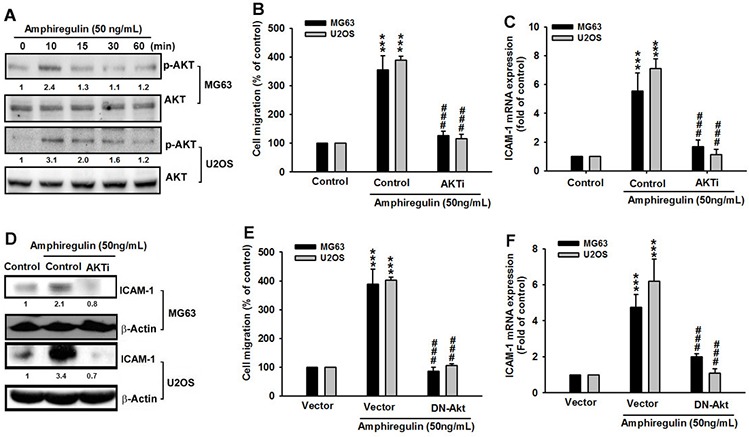
The involvement of Akt in AREG-mediated migration of human osteosarcoma cells **A.** Cells were treated with AREG (50 ng/ml) for the indicated times. The levels of Akt and its phosphorylated form, p-AKT, were determined by Western blotting. **B–D.** Cells were pretreated for 30 min with the Akt inhibitor (Akti) (1 μM) for 1 hr followed by treatment with AREG for 24 hr. The cell migration and ICAM-1 expression were measured. **E–F.** Cells were transfected with dominant negative Akt gene (DN-AKT) for 24 hr followed by treatment with AREG for 24 hr. The cell migration and ICAM-1 expression were measured. Data are shown as the mean ± SEM. The asterisks represent *p* values obtained by *t*-test comparing to what was observed with 0.1% DMSO only (Control). ***represents *P* < 0.001. ##represents *P* < 0.01 for one-way ANOVA comparisons as indicated. ###represents *P* < 0.001.

To further understand whether the downstream target of the PI3K signaling pathway, Akt can also be activated by AREG treatment, we determined the level of phosphorylated Akt at Ser473, which was used in a previous study as an indicator of Akt activity [63]. We determined that AREG treatment increased the phosphorylation of Akt at Ser473 (Figure 4A). Moreover, like its upstream regulator, PI3K, Akt regulates the AREG-induced cancer cell migration of osteosarcoma and the expression of ICAM-1 at treatment with an Akt inhibitor (Akti) [61], can inhibit the AREG-induced migration and ICAM-1 up-regulation (Figure 4B–4D). In addition, we observed the same suppressive effect in the dominant-negative mutant of Akt-transfected cells (Figure 4E–4F). Overall these results imply that AREG and EGFR act through the PI3K/Akt-dependent signaling pathway to enhance ICAM-1 expression and cell migration in human osteosarcoma cells.

### PI3K/Akt mediates AREG-induced ICAM-1 expression via its downstream IKK/NF- κB signaling pathway

**Figure 5:**
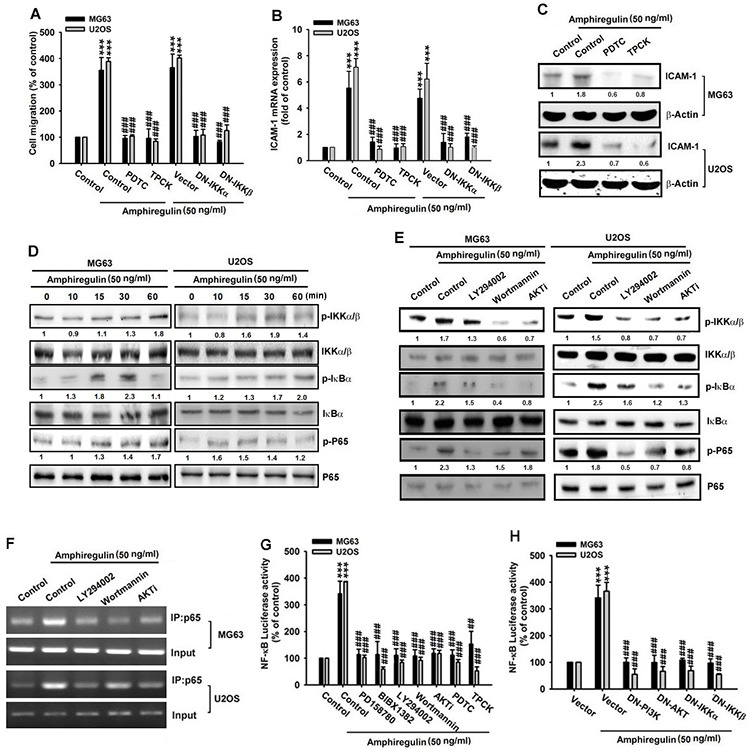
NF-κB mediates the response of human osteosarcoma cells to AREG stimulation **A–C.** Cells were pretreated with 0.1% DMSO as control, PDTC (10 μM) or TPCK (10 μM) for 30 min or were transfected with dominant negative gene of IKKα or IKKβ for 24 hr, followed by AREG treatment for 24 hr. The cell migration and ICAM-1 expression were then measured. **D.** Cells were treated with AREG (50 ng/ml) for the indicated times and the levels of phosphorylated IKKα/β, IκBα, and p65 were examined by Western blotting. **E.** Cells were pretreated with 0.1% DMSO as control, LY294002 (10 μM), Wortmannin (1 μM) or AKTi (1 μM) for 1 hr followed by stimulation with AREG for 30 min. The level of IKKα/β, IκBα, or p65 and their phosphorylated forms were determined by Western blotting. **F.** Cells were pretreated with 0.1% DMSO as control, LY294002 (10 μM), Wortmannin (1 μM) or AKTi (1 μM) for 30 min, followed by AREG treatment (50 ng/ml) for 120 min. Chromatin immunoprecipitation was performed with antibody against p65. One percent of immunoprecipitated chromatin was assayed to verify equal loading (Input). **G–H.** Osteosarcoma cells were transfected with a plasmid harboring the NF-κB luciferase reporter for 24 hr and then the stable cell lines were treated with indicated inhibitors for 30 min or was transfected with different dominant negative genes for 24 hr. Luciferase activity was measured after AREG stimulation for 24 hr. Data are shown as the mean ± SEM. The asterisks indicate *t*-test comparisons to the control of 0.1% DMSO treatment or to cells transfected with the empty vector (Vector). ***represents *P* < 0.001. #represents *P* < 0.05 for one-way ANOVA comparisons as indicated. ##represents *P* < 0.01. ###represents *P* < 0.001.

Nuclear factor-κB (NF-κB) is a major transcription factor that regulates several genes involved in cancer cell migration and invasion [64]. The activation of the factor requires IκB kinase (IKK), a substrate of Akt; thus, the activation of Akt stimulates NF-κB activity. To understand whether NF-κB and IKK are involved in AREG-induced cancer cell migration, we treated osteosarcoma cells with a NF-κB inhibitor(PDTC) and an IKK inhibitor (TPCK). We found that both inhibitors suppressed the AREG-induced cancer cell migration and ICAM-1 expression in mRNA and protein levels (Figure 5A–5C). Furthermore, the expression of dominant-negative mutant of IKKα or IKKβ mimicked the effect of the IKK inhibitor to constrain the AREG-induced cell migration and ICAM-1 mRNA expression (Figure 5A–5B). We further investigated whether AREG activates NF-κB signaling through the PI3K/Akt pathway. We first observed that AREG stimulation increased the phosphorylation of IKKα/β (ser^176/180^), IκBα (ser^32/36^) and NF-κB p65 subunit (ser^536^) in a time-depend pattern (Figure 5D); these kinase, are all known to contribute to NF-κB activation [65]. Moreover, PI3K inhibitors (Ly294002, or Wortmannin) and pan-Akt inhibitor (Akti) reduced the AREG-induced phosphorylation of IKKα/β, IκB and NF-κB p65 (Figure 5E), indicating that the PI3K/Akt pathway modulates the activation of NF-κB in response to AREG stimulation. Notably a decrease of phosphorylated IκBα (ser^32/36^) in the MG63 cell was observed at the 60 min point compared with that at the 30 min point (Figure 5D), possibly because of phosphorylation at ser^32/36^ resulting in its own subsequent ubiquitination and degradation [66].

The data presented in the preceding paragraphs suggest that AREG activates the transcription factor NF-κB which in turn upregulates ICAM-1 expression. Therefore, we can speculate that the NF-κB p65 subunit may bind to the NF-κB element on the ICAM-1 promoter after AREG stimulation. To test this, we assessed *in vivo* recruitment of p65 to the ICAM-1 promoter (−346 to −24) by conducting a chromatin immunoprecipitation assay [15]. We determined that AREG treatment enhanced the association of p65 with the ICAM-1 promoter. However, the PI3K inhibitors (Ly294002 or Wortmannin) and Akt inhibitor (Akti) reduced the AREG-induced binding of p65 to the NF-κB element (Figure 5F). This finding is consistent with those of previous studies, confirming that AREG activates NF-κB signaling to modulate ICAM-1 expression through the PI3K/Akt pathway. Next, we examined the level of the NF-κB-driven transcription by transfecting cells with the NF-κB-luciferase reporter, which enabled us to measure the NF-κB activity. We discovered that NF-κB activity markedly increased upon AREG stimulation, whereas treatments of PD158780, BIBX1382, LY294002, Wortmannin, Akti, PDTC and TPCK blocked this response (Figure 5G). The dominant-negative mutants of PI3K, Akt, IKKα and IKKβ also strongly impaired AREG-induced NF-κB activity (Figure 5H). These results confirm that the PI3K/Akt pathway mediates the response of osteosarcoma cells to AREG, and promotes IKK/NF-κB signaling to induce the expression of ICAM-1.

### Knockdown of AREG expression inhibits cell migration in a mouse model of osteosarcoma

**Figure 6:**
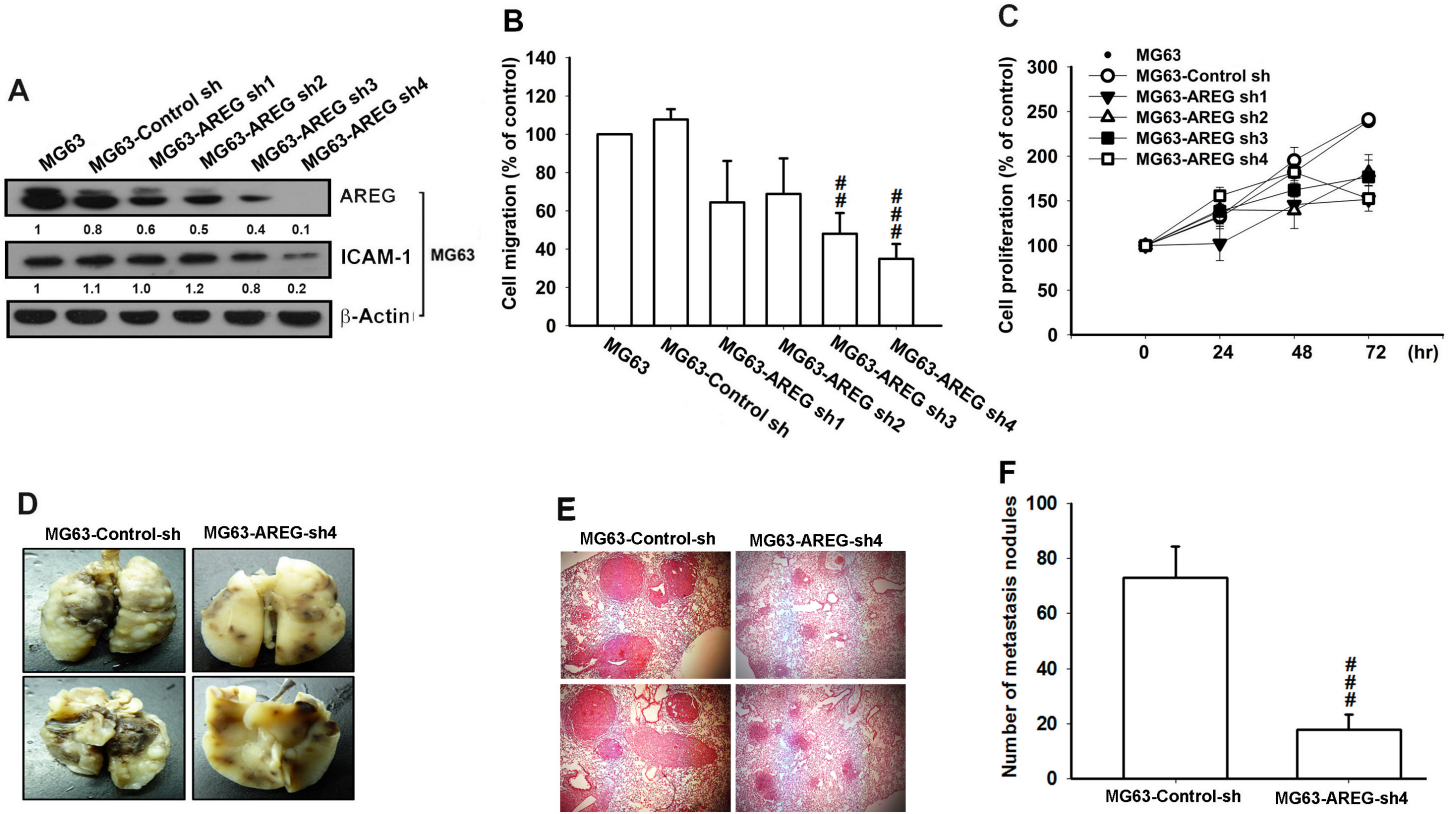
Knockdown of AREG inhibited the migratory ability of osteosarcoma cells **A.** Treating MG63 cells with shRNA against AREG decreased the protein level of AREG as well as that of ICAM-1. **B.** shRNA knockdown of AREG decreased the *in vitro* migration activity of osteosarcoma cells, **C.** but it had no effect on the their cell proliferation rate. **D.** To induce pulmonary metastases, MG63 cells were injected into the mouse tail vein and those mice were sacrificed after 28 days later with developed lung metastatic nodules. Compared to the control mice, there are fewer and smaller tumors which were seen on the lungs of mice injected with osteosarcoma cells transfected with shRNA against AREG. **E.** H&E staining of lung metastatic nodules of MG63 injected mice. **F.** The lungs of MG63 injected mice were removed and inflated with 10% paraformaldehyde fixative. The number of lung metastatic nodules was counted under a dissecting microscope. Results are shown as mean ± SEM. ##represents *P* < 0.01 fort-test comparisons to cells harboring only the empty vector as control. ###represents *P* < 0.001.

According to the results described thus far, we can hypothesize that elevated levels of AREG in osteosarcoma stimulate cancer cell migration by promoting ICAM-1 expression. To test this hypothesis, we reduced the AREG level in the MG63 cells by stably expressing human AREG shRNA. Following puromycin (10 μg/mL) selection, we isolated four individual shRNA clones and compared them with the harboring only the empty vector; we observed that the expression levels decreased in all clones at dissimilar degrees (Figure 6A). We also found that the degree of change in the AREG level is associated with the drop in the ICAM-1 expression and in cell migration rate (Figure 6A–6B). However, the knockdown of AREG did not alter the cell proliferation rate (Figure 6C). We examined the effect of AREG on the osteosarcoma cell migration *in vivo* by injecting MG63 cells (5 × 10^6^) into the tail veins of mice. The mice were sacrificed after 28 days with developed osteosarcoma lung metastases. To investigate whether AREG expression would influence tumor metastasis *in vivo*, we monitored the metastatic potential of the MG63 cells stably expressing control shRNA or AREG shRNA in mouse models of lung metastasis. On day 28 after injection, the tumor size in the lung was significantly reduced when AREG expression was knocked down (Figure 6D). Next, we applied histological analyses to lung tissues from mice by using hematoxylin and eosin (H&E) staining. The lung tissues from mice injected with AREG knockdown MG63 cells exhibited a nearly normal structure or dramatically reduced degrees of lung metastatic nodules. However, lung tissues from the control group mice were heavily infiltrated (Figure 6E). To further investigate the metastatic potential of osteosarcoma defined by counting the numbers of metastatic nodules, the mean number was significantly decreased in the mice injected with AREG shRNA expressing cells (Figure 6F), strongly indicating that AREG promotes cell metastasis and tumor progression of osteosarcoma *in vivo*.

## DISCUSSION

Although osteosarcoma is an uncommon tumor, it is the primary malignant bone tumor prevalent in children and adolescents. The survival rate of osteosarcoma has improved immensely with the inducetion of chemotherapy. Nonetheless, despite achieving local tumor control, 80% of patients with osteosarcoma develop metastatic disease. ICAM-1 is a cell surface transmembrane glycoprotein, which is associated with several inflammatory and immune responses. ICAM-1 is an adhesion molecule that is involved in cancer metastasis. The effect of AREG on tumor migration has been explained previously. The overexpression of growth factor receptors of tumor cells, which enhance the cell's downstream signaling, increase their proliferation rates and metastatic potential in addition to promoting angiogenesis. Willmarth and Ethier reported that MCF10A cells overexpressing AREG exhibited increased cell motility and invasion capabilities and that AREG up-regulated the expression of several genes associated with cell adhesion and motility [33]. Moreover studies have reported that most malignant breast cancer cell lines express a high level of AREG, suggesting that AREG may promote metastasis [67, 68]. In addition to our findings, several studies have demonstrated that AREG activates EGFR signaling to synthesize, secrete, and activate proteins involved in invasion and metastasis such as urokinase-type plasminogen activator (uPA), matrix metalloproteinase 9 (MMP-9), and extracellular matrix metalloproteinase inducer (EMMPRIN) [69–71].

Immunohistochemistry (IHC) applied for clinical specimens derived from patients with osteosarcoma revealed that the AREG expression level correlated with tumor stage in osteosarcoma. Using cellular-level experiments, we also demonstrate that AREG promotes ICAM-1 expression and migration in osteosarcoma. In addition, AREG promotes ICAM-1 expression and migration via EGFR signaling through the PI3K/Akt/NF-κB pathways. Therefore, AREG may be a new molecular therapeutic target for reduction of metastasis in osteosarcoma.

Increasing evidence suggests that EGFR pathways are involved in the progression of cancer of the bone, soft tissue, breast, and lungs [72, 73]. EGFR expression is linked to poor prognosis in numerous cancers. In addition, EGFR inhibitor gefitinib (Iressa) is successful for treating of non-small cell lung cancer [74]. EGFR expression has even been proven to predict poor outcome in osteosarcoma [75, 76]. Here, we report that treatment with an EGFR inhibitors or siRNA antagonized AREG-induced ICAM-1 expression and cell migration. Incubation of osteosarcoma cells with AREG promotes the level of phosphorylated EGFR at tyrosine 1068 and 992. These results indicate that AREG and EGFR interact together to regulate the migration of osteosarcoma and the expression level of http://ICAM-1.In this study, we conducted a detailed analysis of AREG association with osteosarcoma metastasis. In summary, we determined that: 1) two osteosarcoma cell lines expressed high levels of AREG; 2) exogenous AREG further enhanced cell migration of osteosarcoma cells and the expression of ICAM-1; 3) these AREG-induced enhancements were suppressed by siRNA knockdown of ICAM-1 or EGFR, with the same suppressive effect mimicked by treating with inhibitors of EGFR, PI3K, Akt, NF-κB, and IKK or by expressing dominant-negative mutant forms of PI3K, Akt, IKKα, and IKKβ; 4) exogenous AREG also increased the activities of EGFR, PI3K, Akt, and NF-κB by enhancing their phosphorylated levels; 5) the AREG increased-activity of NF-κB can be abolished by treatment with inhibitors of EGFR, PI3K, Akt, NF-κB, and IKK, or by expressing dominant-negative mutant forms of PI3K, Akt, IKKα, and IKKβ; 6) AREG stimulation promoted the association of NF-κB to the ICAM-1 promoter, which then up-regulated the ICAM-1 expression; 7) osteosarcoma cells over-expressing AREG shRNA exhibited less motility, and mice injected with these cells demonstreated fewer and smaller metastatic nodules.

**Figure 7:**
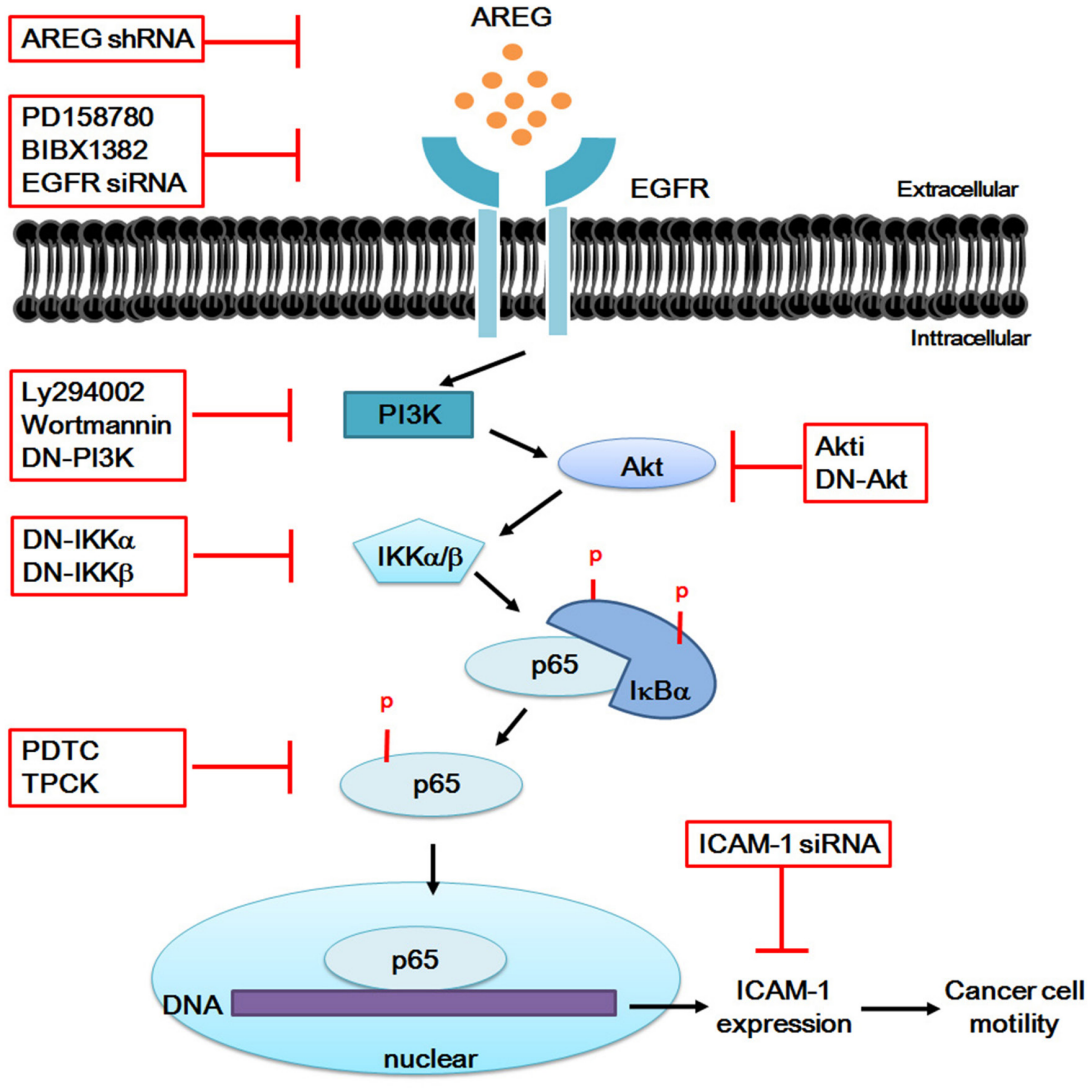
Schematic diagram illustrating the proposed signaling pathway involved in AREG-induced ICAM-1 expression in human osteosarcoma cells AREG induces the activation of NF-κB p65 through EGFR/PI3K/Akt signaling pathway. Activated NF-κB p65 was recruited to the promoter region of ICAM-1 leading to an increased expression of ICAM-1. Therefore, blockade of these pathways by treatments of different inhibitors or RNAi knockdown can reduce ICAM-1 expression and then decrease the tumor cell motility of osteosarcoma cells. See “Discussion” for further explanation.

In summary, our findings suggested that AREG promoted cancer cell motility of osteosarcoma and up-regulated the expression of ICAM-1 through the EGFR/PI3K/Akt/NF-κB signaling pathway (Figure 7). Genetic silencing through transfection with siRNA of EGFR or ICAM-1 and pretreatment with inhibitors of EGFR, PI3K, Akt, NF-κB and IKK abrogated the AREG-enhanced ICAM-1 expression and cancer cell migration. However, exogenous AREG enhanced cell migration and ICAM-1 expression. Our findings indicated that AREG plays a critical role in tumor invasion and tumorigenesis of bone sarcomas. Indeed, studies have reported that AREG stimulates directional migration and invasion of human cancer cells [43, 77]. Moreover, we observed that overexpression of AREG shRNA inhibited cancer migratory ability by approximately 60% (Figure 6B). Overall, we validated that the expression of AREG is associated with osteosarcoma metastasis, signifying that AREG is a novel marker for cancer progression and metastasis of bone sarcomas.

## MATERIALS AND METHODS

### Materials

Protein A/G beads, anti-mouse and anti-rabbit IgG conjugated horseradish peroxidase and polyclonal antibodies that are specific for AREG, VCAM-1, EGFR, PI3K, Akt, IKKα/β, IκB, p65 and β-Actin were purchased from Santa Cruz Biotechnology (Santa Cruz, CA, USA). Polyclonal antibodies that are specific for EGFR phosphorylated at PY^1068^ or at PY^992^, PI3K phosphorylated at Tyr^458/199^, Akt phosphorylated at Ser^473^, IKKα/β phosphorylated at Ser^176/180^, IκBα phosphorylated at Ser^32/36^, p65 phosphorylated at Ser^536^ and ICAM-1 were purchased from Cell Signaling and Neuroscience (Danvers, MA, USA). PD158780, BIBX1382, PDTC (Pyrrolidine-dithiocarbamate), TPCK (L-1-tosylamido-2-phenylenylethyl chloromethyl ketone), LY294002, Wortmannin, and AKT inhibitor (1L-6-hydroxymethyl-chiro-inositol-2-(*R*)-2-*O*-methyl-3-*O*-octadecylcarbonate) were purchased from Calbiochem (San Diego, CA, USA). The recombinant human AREG was purchased from PeproTech (Rocky Hill, NJ, USA). The NF-κB luciferase plasmid was purchased from Stratagene (La Jolla, CA, USA). The p85 and Akt (Akt K179A) dominant-negative mutants were gifts from Dr. Wen-Mei Fu (National Taiwan University, Taipei, Taiwan). The IKKα (IKKα-KM) and IKKβ (IKKβ-KM) dominant-negative mutants were gifts from Dr. H. Nakano (Juntendo University, Tokyo, Japan). The pSV-β-galactosidase vector and the luciferase assay kit were purchased from Promega (Madison, MA, USA). Unless otherwise specified, all other chemicals were obtained from Sigma-Aldrich (St. Louis, MO, USA).

### Cell culture

The human osteosarcoma cell lines (U2OS and MG63) and human fetal osteoblastic cell line (hFOB 1.19) were purchased from the American Type Cell Culture Collection (Manassas, VA, USA). Cells were maintained as previously described [78–80]. All experiments with hFOB 1.19 cells were carried out at the permissive temperature of 33.5°C for the expression of the large T antigen.

### Cell proliferation assay

Cell proliferation assay was performed using MTT (3-(4, 5-dimethylthiazol-2-yl)-2, 5-diphenyl tetrazolium bromide) (Sigma-Aldrich, St. Louis, MO, USA). Cells were seeded in a 96 well plate (1 × 10^4^ cells/well). After 24 hr, the cells were subjected to the indicated treatments for 24 hr. Cell proliferation was measured by adding 10 μl of MTT stock solution (5 mg/mL) to each well, and the plate was incubated at 37°C for 2 hr. To solubilize formazan, dimethyl sulfoxide (DMSO) was added, and the plate was incubated in a humidified chamber at 37°C for 30 min. The cell proliferation was then determined by measuring the absorbance at 570 nm which can be read using a microplate spectrophotometer (PowerWave X340, BioTek Instruments, Winooski, VT, USA).

### Migration assay

Using Transwell^®^, the migration assay was measured according to a previously described method that was modified as following [81]. Before performing the migration assay, cells were treated for 30 min with different concentrations of inhibitors (LY294002, Wortmannin, Akt inhibitor, PDTC or TPCK) or their solvent control (0.1% dimethyl sulfoxide). Approximately 1 × 10^4^ cells in 200 μl of serum-free medium were placed in the upper chamber and 300 μl of the same medium containing different concentrations of AREG were placed in the lower chamber. The cells were incubated at 37°C with 5% CO_2_ for 24 hr, and then were fixed with 3.7% formaldehyde for 15 min and stained with 0.05% crystal violet in phosphate-buffered saline (PBS) for 15 min. Cells were counted under an OLYMPUS IX70 inverted phase-contrast microscope. Each experiment was performed in triplicate and was repeated at least three times. The number of migrating cells in each experiment was adjusted by the cell viability assay to correct for proliferation effect of AREG treatment (corrected migrating cell number = counted migrating cell number/percentage of viable cells).

### Wound-healing migration assay and western blot analysis

Wound-healing migration assay and western blot analysis were performed as previously described with following modifications [46, 82]. After transfer, the blots were blocked with 5% BSA at room temperature for 1 hr. and then probed with different rabbit anti-human antibodies that are against PI3K, Akt, IKKα/β, IκB, p65, EGFR, ICAM-1 (1:1000) at room temperature for 1 hr. The blots were visualized by enhanced chemiluminescence which can be detected by FUJI Super RX-N X-RAY film (Fujifilm Corporation, Tokyo, Japan). Quantitative data were obtained using a computing densitometer and Image Quant software (Molecular Dynamics, Sunnyvale, CA, USA).

### Quantitative real time PCR

Total RNA was extracted from the cells using a TRIzol kit (MDBio Inc., Taipei, Taiwan) and then 2 μg RNA was used for synthesis of complementary DNA (cDNA) by reverse transcriptase (Invitrogen, Carlsbad, CA, USA). Quantitative real-time PCR was carried out using SYBR Green (KAPA Biosystem, Woburn, MA, USA) according to the manufacturer's protocol and reactions were run on the StepOnePlus^TM^ machine (Applied Biosystems, Foster City, CA, USA). The reaction conditions were 10 min at 95°C for polymerase activation and 40 cycles of 15 s at 95°C and 60 s at 60°C. The following primers were used to amplify target genes: human ICAM-1 forward (5′-GCAC ATTGGTTGGCTATCTTCT-3′), ICAM-1 reverse (5′-GCC CGAAGCGTTTACTTTGA-3′), human VCAM-1 forward (5′-GGGACCACATCTACGCTGACA-3′), VCAM-1 reverse (5′-CCTGTCTGCATCCTCCAGAAA-3′), human GAPDH forward (5′-AGGGCTGCTTTTAACTCTGGT-3′), GAPDH reverse (5′-CCCCACTTGATTTTGGAGGGA-3′) [83, 84]. The expression levels of ICAM-1 or VCAM-1 were determined by normalizing to that of GAPDH. The threshold cycle (Ct) was set above the non-template control background and within the linear phase of amplification of target genes in order to calculate the cycle numbers at which the transcript was detected (denoted Ct). Each sample was assayed in triplicate and the data shown are representatives of three independent experiments.

### Transient transfection of small interfering RNA (siRNA)

siRNA of human ICAM-1 and EGFR as well as a non-targeting siRNA as a negative control were purchased from Santa Cruz Biotechnology (Santa Cruz, CA, USA). Transient transfection of siRNA (100 nM) was performed using Lipofectamine 2000 Reagent (Invitrogen, Carlsbad, CA, USA) according to the manufacturer's instructions.

### Transfection and reporter gene assay

Human osteosarcoma cells were co-transfected with 0.8 μg NF-κB driven luciferase plasmid and 0.4 μg β-galactosidase expression vector. Cells were grown to 80% confluence in 12-well plates and were transfected on the following day by Lipofectamine 2000 (LF2000; Invitrogen). DNA and LF2000 were premixed for 30 min and then were applied to the cells. DMEM containing 20% FBS was added 4 hr later. After 24 hr of transfection, the cells were treated with the indicated agents. After further 24 hr incubation, the media were removed and cells were washed with cold PBS. To prepare lysates, 100 μl reporter lysis buffer (Promega, Madison, WI) were added to each well and cells were scraped from dishes. The supernatant was collected after centrifugation at 11,000 *g* for 2 min. Aliquots of cell lysates (20 μl) containing equal amounts of protein (20–30 μg) were placed into wells of an opaque black 96-well microplate. An equal volume of luciferase substrate was added to all samples and luminescence was measured in a microplate luminometer. The level of luciferase activity was normalized to transfection efficiency monitored by the co-transfected β-galactosidase expression.

### Chromatin immunoprecipitation (ChIP)

ChIP was performed as described [85]. DNA was immunoprecipitated using an anti-p65 antibody and then was extracted, purified, and resuspended in H_2_O. Immunoprecipitated DNA was used as template for PCR using the following primers which are specific for the ICAM-1 promoter: 5′-AGACCTTAGCGCGGTGTAGA-3′ and 5′-GCGACTCGAGGAGACGATGA-3′ [15]. PCR products were resolved by 1.5% agarose gel electrophoresis and visualized by UV light.

### Establishment of stably transfected cells

The AREG and control shRNA lentiviral constructs (pLKO.1) were obtained from the National RNAi Core Facility (Academia Sinica, Taipei, Taiwan). These two constructs were individually transfected into HEK293T cells along with packaging vectors, pCMV and pMDG. The cell culture supernatants containing lentiviruses were harvested at 24 hr and 48 hr post-transfection and stored at −80°C. The MG63 cells were infected with viral supernatants in the presence of 8 μg/ml of polybrene (Sigma–Aldrich). After 48 hr of infection, the cells were treated with puromycin (10 μg/ml) for selection. The selection medium was replaced every 3 days. After 2 weeks of selection with puromycin, clones of resistant cells were isolated.

### *In vivo* tumor xenograft study

All animal experiments were performed in accordance with a protocol approved by the Institutional Animal Care and Use Committees (IACUC) of Shin-Kong Wu Ho-Su Memorial Hospital (Taipei, Taiwan). Male CB17-SCID mice 4-weeks old were used. To assay the metastatic potential of osteosarcoma, 5 × 10^6^ MG63 transfected cells were resuspended in 0.1 ml of saline and were injected into the tail vein. After injection, visible macroscopic pulmonary metastases were present by 4 weeks and those mice were euthanized by an overdose of the anesthetic agent. The lungs were removed and fixed in 10% paraformaldehyde. The number of lung tumor metastases was counted under a dissecting microscope.

### Statistical analysis

Statistical significance between two samples was determined by using Student's *t*-test. Statistical comparisons of more than two groups were performed using one-way ANOVA with Bonferroni's *post hoc* test.

## SUPPLEMENTARY FIGURE


