# Supplementary material for: Amphiregulin enhances intercellular adhesion molecule-1 expression and promotes tumor metastasis in human osteosarcoma
Source: Oncotarget. 2015 Oct 19;6(38):40880–95. doi: 10.18632/oncotarget.5679 (PMC4747375; doi:10.18632/oncotarget.5679)
Supplement: Supplementary file 1 [file oncotarget-06-40880-s001.pdf]

SUPPLEMENTARY FIGURE

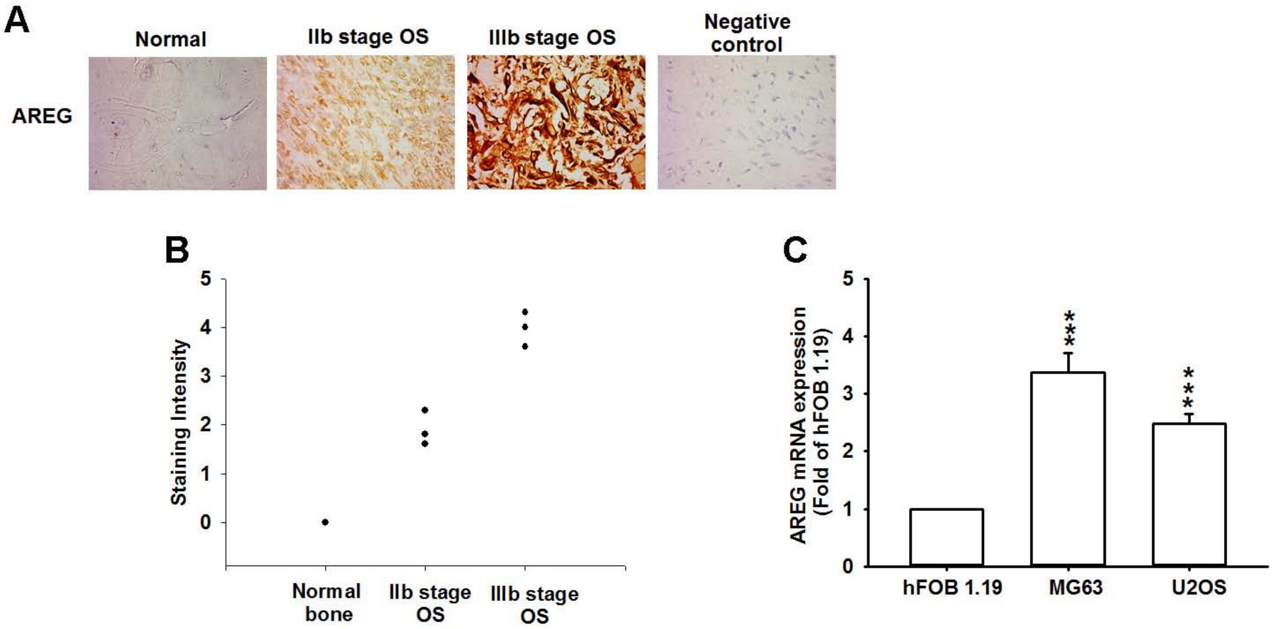

Supplementary Figure S1: The level of AREG between the normal and the malignant cells. A–B. Tumor specimens were immunostained (IHC) with anti-AREG antibody. The staining intensity was scored 0–5. **C.** Total RNA was extracted from hFOB 1.19, MG63, and U2OS cells and the levels of AREG and ICAM-1 were examined by qPCR. All bars represent the mean ± SEM. The asterisks indicate that the data are significantly different from the hFOB 1.19.
